# Merkel Cell Carcinoma: When Does Size Matter for Radiotherapy?

**DOI:** 10.7759/cureus.443

**Published:** 2015-12-31

**Authors:** Farshad Kasraei, Michelle Roach, Mark T Lee

**Affiliations:** 1 Radiation Oncology, Liverpool Hospital, NSW; 2 Cancer Therapy Centre, Liverpool Hospital, NSW; 3 Medicine, University of New South Wales

**Keywords:** radiotherapy, merkel cell carcinoma

## Abstract

Merkel cell carcinoma is a very aggressive, rare cancer of the skin. It has a high propensity for local, regional, and distant recurrence and has recently been associated with a viral etiology from the recently diagnosed Merkel Cell Polyoma Virus. The optimal management remains controversial. We discuss the case of a man with a 26 cm axillary lymph node metastasis of unknown primary treated with radiotherapy.

## Introduction

Merkel cell carcinoma (MCC) is a rare neuroendocrine skin cancer that has been described as the most aggressive cutaneous malignancy. The cell of origin is Merkel cell or skin pressure receptor, which has a tendency for dermal lymphatic invasion, nodal spread, and distant metastatic spread. Exposure to sunlight and immunosuppression are some of the factors that have been implicated in its cause as well as an association with the recently found Merkel Cell Polyomavirus (MCPyV). The immune response appears to have a significant impact on MCC with spontaneous regression of the primary tumor having been documented and up to 25% of patients presenting with no obvious primary tumor [1-4]. These MCCs of unknown primary are particularly challenging because of the potential for late metastatic disease in unusual regions of the body. Recent literature suggests that patients with the presence of an occult primary tumor are associated with a more favorable prognosis. MCC, being a high-grade neuroendocrine carcinoma, has a response similar to small-cell carcinoma of the lung. It has high response rates to radiotherapy and chemotherapy. However, management of patients with MCC is still a challenge for the clinician, particularly with respect to the optimal choice for local and systemic management [1-2, 5-7].

## Case presentation

A 55-year-old man was referred for management of a 26 cm diameter lymph node metastasis from MCC in the right axilla. He had a long history of sun exposure, as his main occupation was grass mowing, but he did not have a history of previous skin cancer. He was managed five months prior to his referral to the cancer center for a suspected infected right axillary abscess. Persistence of the mass was thought to be due to a hematoma; however, two further aspirations were unsuccessful. The collection continued to reform with persistence of purulent discharge, and he was treated with antibiotics. Two months later, the mass had significantly enlarged. He had symptoms of local discomfort as well as paresthesias in his right upper arm and thumb and weakness of his right thumb opposition and flexion. A core biopsy of the mass was performed which confirmed the nature of the malignancy with strong positive immunoreactivity to neuroendocrine carcinoma markers, CD56, chromogranin A, and synaptophysin. Other stains, including Cytokeratin 20 (CK20), CK7, CD45, S100, Melan A, thyroid transcription factor 1 (TTF1), and calcitonin were negative. The Ki-67 proliferative index was more than 90%.

To stage his cancer, a CT scan of his chest, abdomen, and pelvis was performed, which showed a heterogeneous 16 x 15 x 14 cm right axillary mass and surrounding enlarged lymph nodes. A heterogeneous 4.6 x 3.7 x 6.0 cm left thyroid lobe was also seen, extending into the upper mediastinum in a retrosternal location with further enlarged mediastinal lymphadenopathy involving the left paratracheal and paraesophageal lymph nodes. As the CT scan was not conclusive of metastatic disease, an 18 fluoro-deoxy-glucose – positron emission tomography (FDG-PET) scan was organized to exclude distant metastatic disease. His anterior chest wall skin was erythematous and edematous; therefore, punch biopsies of this area were performed, which showed edema but no dermal lymphatic invasion. His PET scan was performed with a slightly elevated glucose level and showed mild increased FDG uptake in the large axillary mass, but no uptake in the thyroid and no evidence of metastatic disease. His case was discussed at a multi-disciplinary team meeting with members from nuclear medicine, radiology, pathology, surgery, and oncology. The negative TTF-1 staining and lack of FDG-PET uptake in the thyroid and lung were felt to exclude both lung and thyroid as the primary for the cancer and to be in keeping with a MCC stage T0N1M0 (Stage IIIB). Although CK20 stain is typically (but not always) positive in MCC, the location of the lymph node disease in the axilla was felt to be in keeping with a likely primary that had potentially regressed from the skin of the arm or the chest and back. The recommendation was that he should receive semi-urgent definitive radiotherapy with or without chemotherapy. As he had an open sinus and had previously infected collections; chemotherapy was determined to be unsafe to administer due to the risk of neutropenic sepsis.

**Figure 1:**
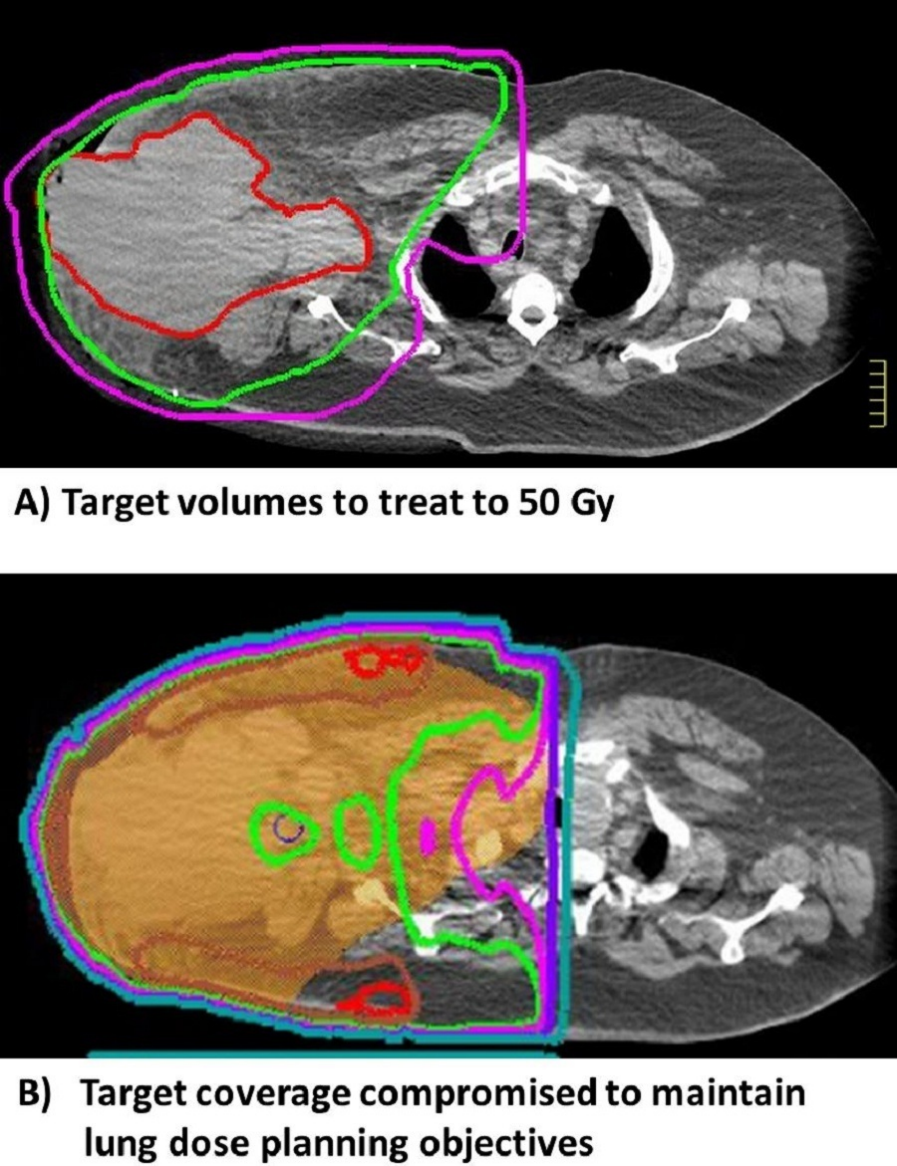
Target volumes and coverage A) Target volumes to treat tumour. Red contour is the GTV, Green is the CTV and Magenta is the PTV. B) Shows the dose coverage with the orange colourwash showing the adjusted PTV to limit lung exposure. Red:55Gy, Brown:53.5 Gy, Green:50 Gy, Pink:47.5 Gy, Purple:40 Gy and Teal:20 Gy.

One week after his CT scan, at his first review by the radiation oncology team, the mass measured about 26 cm in the anterior and posterior directions with a 6-8 cm ulcerated skin nodule growing through the previous drain site. He was started on dexamethasone to help reduce his brachial plexopathy. Due to the size of his disease, the radiation oncologist planned for him to receive 60 Gy of radiation in 2 Gy fractions.The planned doses were for the 50 Gy clinical target volume (CTV) to cover the right axilla, chest wall, and draining infra and supra-clavicular lymph node regions and the 60 Gy to cover the gross disease with a 2-3 cm margin. A 1 cm margin was used to allow for geometric uncertainty to make planning target volume (PTV) (Figure 1). He was positioned supine with his hands akimbo for radiotherapy simulation in a large vacuum immobilisation bag, and 15 mm of customized bolus was used to ensure that 100% of the dose would be received to the skin. Conventional radiotherapy (as compared to intensity modulated radiotherapy) was felt to offer the most robust treatment to account for geometric uncertainty from his large area of bolus placement and potential shrinkage of the cancer through treatment. His PTV width was 39 cm at maximum dimensions, and he was treated with an extended focus-source distance (FSD) length to allow appropriate coverage of the PTV. His radiotherapy planning CT scan was performed on a Friday, and he started treatment the following Tuesday with a simple anterior/posterior opposed beam technique using 18 MV photon beams to improve dose heterogeneity, although fairly large doses >110% of the prescription dose were seen on this phase of treatment. The main dose constraints aimed for a lung V20 of < 30% and mean lung dose < 20 Gy. The planned dose for the opposed beam technique provided a V20 of 49% and a mean lung dose of 22.8 Gy. Therefore, a second phase was developed to cover the 50 Gy CTV volume after 16 Gy, specifically to reduce the lung volume being irradiated. The CTV 50 Gy was slightly reduced in size, and the PTV 50 Gy volume coverage was slightly compromised to maintain acceptable lung DVH for the 34 Gy in 2 Gy fractions to the elective lymph node basin. The lung V20 was 10% and a mean lung dose of 6 Gy if treated to 50 Gy for this phase. The summed V20 was 24% with a mean lung dose of 12.8 Gy for the combined phases of both plans, which were considered acceptable.

**Figure 2:**
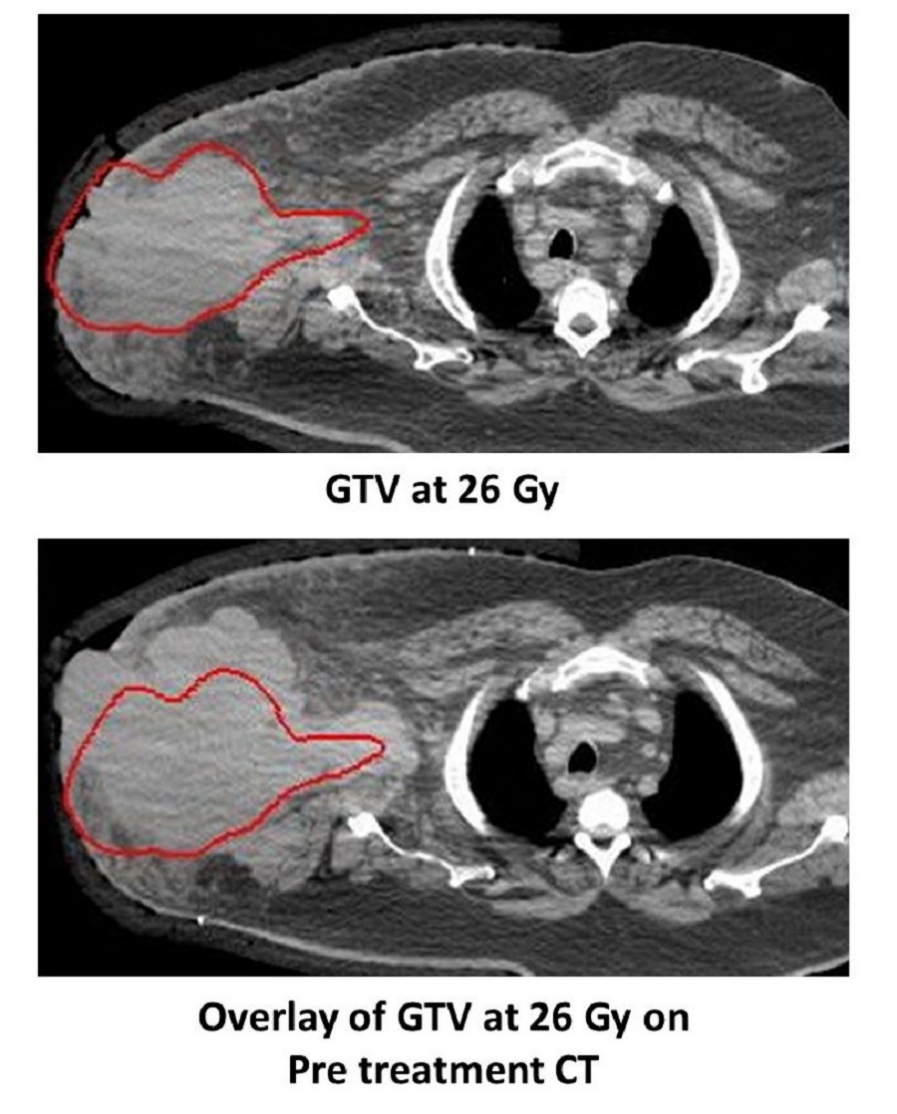
Change in GTV volume at 26 Gy

During his treatment, the tumor reduced substantially in size. He had a replanning CT scan performed at 26 Gy of treatment, and his GTV had reduced in size from 2,552 mL to 1,290 mL (Figure 2). His final phase of radiotherapy (10 Gy boost to the GTV) was planned on this scan. At the fifth week of treatment (54 Gy), he developed a severe Grade 3 acute skin desquamation within his irradiated field with about five to ten small areas of contact bleeding with a nearly complete resolution of his fungating tumor at the drain site. Additionally, he had moderate pain affecting the movement of his arm which required opioid analgesia, and therefore, his radiotherapy was stopped as he had reached his acute toxicity threshold for treatment, completing his course of radiation therapy at 54 Gy. His paraesthesia of his right thumb improved, the thumb flexion improved to normal, and his dexamethasone was stopped at the end of treatment.

**Figure 3:**
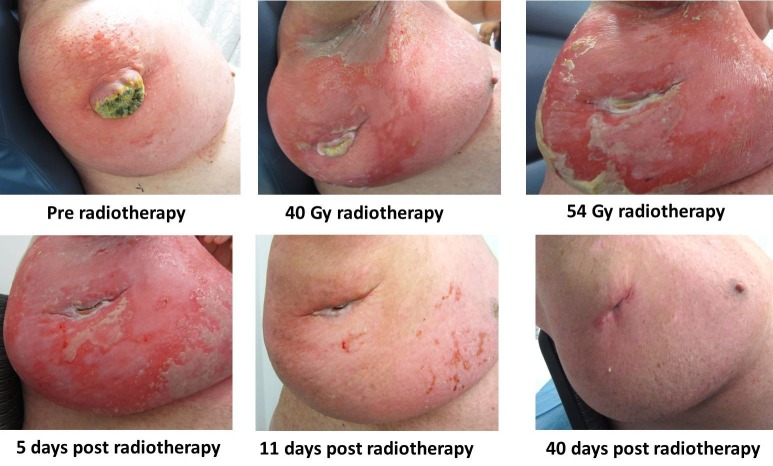
Tumour response from radiotherapy and improvement of skin acute toxicity

As his tumor continued to reduce in volume, the sinus at the drain site continued to increase in size leaving an open cavity in his axilla. For the weeks following treatment, he was managed with metronidazole and Intrasite dressings to the tumor site and sinus, and Intrasite with Jelonet dressings were applied to the remaining areas of skin desquamation. The sinus in the treatment field remained open and had intermittent discharge. Within four weeks of the completion of his radiotherapy, his skin healed completely, but the sinus persisted. Figure 3 shows the clinical response of the mass to treatment and subsequent acute radiation reaction and resolution after treatment.

One month after completing RT, a restaging CT scan showed an 18 x 22 mm right axillary mass and no radiological evidence of distant metastasis. Due to the persistence of the discharging sinus, adjuvant chemotherapy was not started due to the risk of infection, but an axillary dissection was organized to remove the sinus and collection in the axilla. Therefore, two months following his radiotherapy, he underwent a right axillary dissection of levels 1-3 and resection of a large soft-tissue mass and overlying sinus. His histopathology showed two small areas (6 mm) of residual neuroendocrine carcinoma with large areas of necrosis which were located around the sinus tract. He had clear resection margins, and ten lymph nodes found in the adjacent fat had varying degrees of fibrosis, calcification, and focal granulomatous reaction suggestive of tumor regression in at least four of these nodes. Although adjuvant chemotherapy was being considered, the patient’s drain came out accidentally; he subsequently developed a sinus with further purulent discharge without any evidence of arm lymphedema. Chemotherapy was deemed unsafe to start due to the risk of worsening infection.

**Figure 4:**
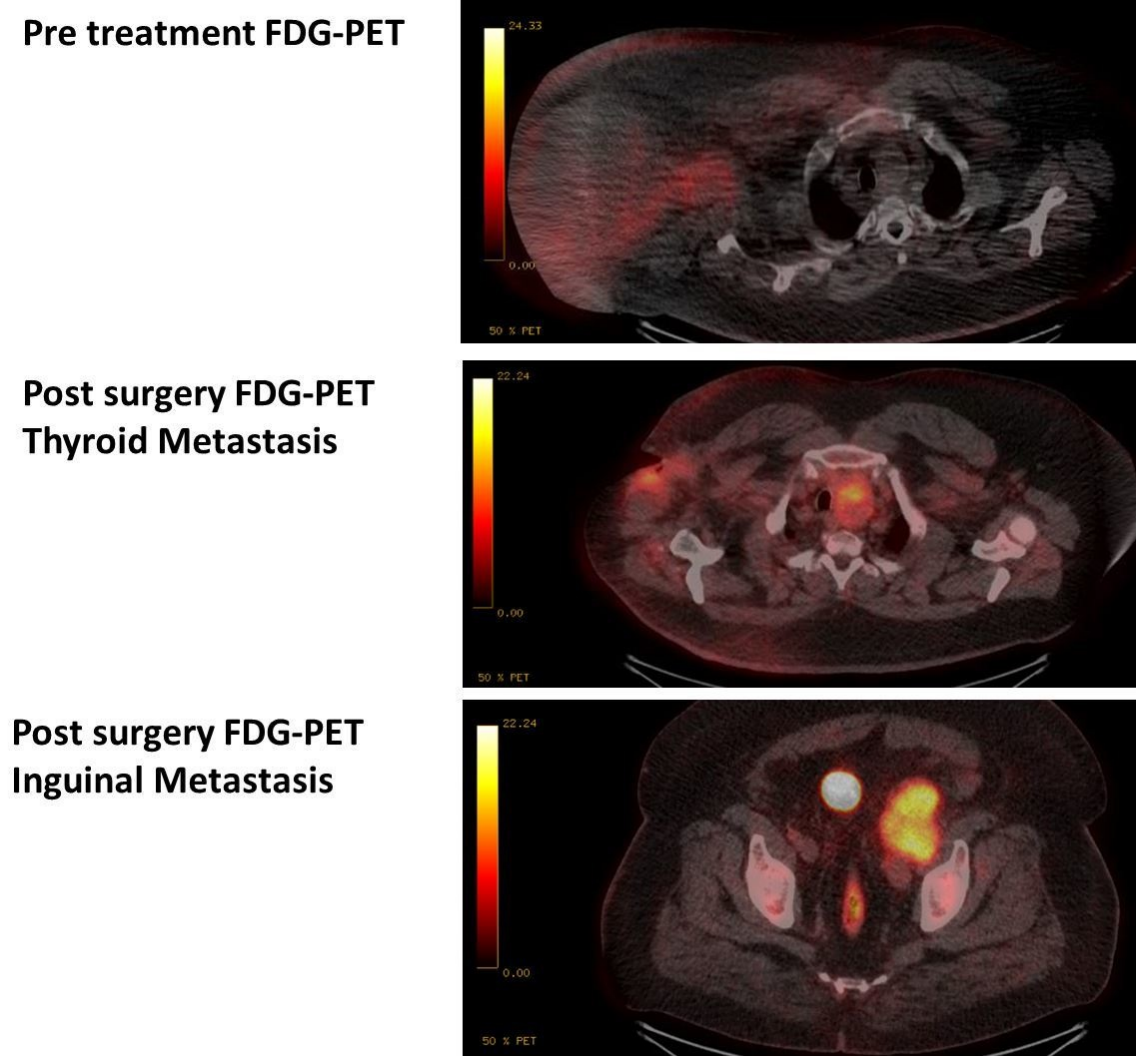
FDG-PET uptake prior to treatment and at the time of diagnosis of distant metastatic disease

Five months after radiotherapy, he had two sinuses in the right axilla--one at the apex of the scar and the other at the posterior axillary line in the skin which had developed due to ongoing serous discharge. His skin was indurated, but with no obvious malignancy. A CT scan was performed and showed no metastases, but did show a persistence of the mass in his left lobe of the thyroid (which initially was not avid on pretreatment FDG-PET scan and was on the opposite side to his axillary nodal disease). An FNA of the thyroid nodule was performed which showed cytology consistent with a metastatic MCC. At this time, a restaging FDG-PET showed no infield loco-regional disease, but there was evidence of new sites of disease, including the left thyroid lobe and the left external iliac lymph node region (Figure 4). Uptake in right axilla and mediastinal lymph nodes were likely to be reactive or inflammatory. A brain CT scan was performed and showed no evidence for brain metastases. He was subsequently started on palliative chemotherapy with carboplatin and etoposide.

## Discussion

MCCs with an occult primary lesion present a diagnostic and therapeutic dilemma. In the MCC, diagnosis is suggested by neuroendocrine features on pathology, and differentiation from small-cell lung cancer is made by negative thyroid transcription factor (TTF) staining and positive staining with CK20. Although our patient had negative staining to CK20, the pathologist and multi-disciplinary team felt that this diagnosis was consistent with Merkel cell carcinoma due to the location of the nodal metastasis and lack of other known primary lesions. Negative TTF-1 staining of neuroendocrine cancers is typically used to exclude lung and thyroid primary lesions. There is no clear consensus about the best treatment option for MCC due to the rarity of this tumor and the concomitant scarce reports of large series of patients with unknown primary. The current literature suggests that the optimum treatment for MCC is a combination of surgery, plus RT in terms of recurrence; however, the impact of multi-modality therapy on distant metastatic disease recurrence is less clear. Additionally, MCC has recently been found to be associated with the MCPyV, which is present in the majority of MCCs in North America; however, the prevalence is less common in Australasia potentially due to heavy sun exposure being another pathway to develop MCC. The immune response potentially mediates the response of MCC to treatments and risk of disease progression. Patients with an unknown primary have been reported to have a better prognosis potentially due to an immune-mediated response and regression of the primary lesion [5-8]. Whether MCC associated with MCPyV has a similar prognosis and response to treatment compared with the sun exposure-related MCC has yet to be confirmed in trials, but may affect the future management of this cancer.

In the case presented, it was not clear that surgery would be feasible due to the proximity of the lymph node disease with the brachial plexus and vessels. In this setting, curative radiotherapy alone was considered. For bulky disease, we aimed for 60 Gy with or without chemotherapy. Given the size of this lymph node disease and potential hypoxia, we felt that regional control, even at this dose, may not have been possible. As surgery was required to close the sinus, the impression was that a comprehensive, axillary lymph node dissection should be performed, although this would increase the risk of lymphedema. Surprisingly, this patient had only a few microscopic areas of residual cancer located around the sinus tract, and it is not clear if these areas were viable or were going to undergo cell death, although hypoxia around this area may potentially have reduced the radiotherapy therapeutic ratio.

As there is no randomised trial, there is a paucity of best evidence with regard to dose and fractionation of radiotherapy; however, an Australian experience of 43 patients showed that patients with MCC treated with RT have a high likelihood of obtaining in-field control. They recommended doses of 50 to 55 Gy in 20 to 25 fractions. A minority of patients were cured, although many died from systemic recurrence; in this study, in-field control rate was 75% [9]. Another retrospective study of 112 patients showed 21 patients had the gross nodal disease of which 11 patients had an occult primary. They suggested that there is dose response for nodal disease. The median dose for the nodal gross disease was 51 Gy (range: 42–65 Gy). The rate of in-field relapse showed a marked decline with every increase of 5 Gy over the range of doses [10].

In this case, the patient was initially planned for 60 Gy in 30 fractions of radiotherapy, but as he developed significant skin toxicity, radiotherapy was stopped at 54 Gy. The volume of skin irradiated is directly related to the risk of dose-limiting toxicity, and, in this case, a multiple phase plan was developed to reduce toxicity. Stopping his radiotherapy early meant that he recovered well from the acute skin desquamation and had completely re-epithelialized the skin within one month after treatment. At the one year follow-up, he had no evidence of loco-regional recurrence and no evidence of late effects. Unfortunately, he developed distant metastasis to the iliac lymph nodes and thyroid, which is a hallmark of this disease.

## Conclusions

In this case, treatment of an enormous nodal Merkel cell carcinoma to a dose of 54 Gy caused an exceptional response to radiotherapy with no loco-regional recurrence in the one-year follow-up; however, the patient developed distant metastasis.

## References

[1] Erovic I, Erovic BM (2013). Merkel cell carcinoma: the past, the present, and the future. Journal of skin cancer.

[2] Poulsen M (2004). Merkel cell carcinoma of the skin. Lancet Oncol.

[3] Feng H, Shuda M, Chang Y, Moore PS (2008). Clonal integration of a polyomavirus in human Merkel cell carcinoma. Science.

[4] Agelli M, Clegg LX, Becker J.C, Rollison DE (2010). The etiology and epidemiology of merkel cell carcinoma. Curr Probl Cancer.

[5] Huber GF (2014). Modern management of Merkel cell carcinoma. Curr Opin Otolaryngol Head Neck Surg.

[6] Kontis E, Vezakis A, Pantiora E, Stasinopoulou S, Polydorou A, Voros D, Fragulidis GP (2015). Merkel cell carcinoma of unknown primary site; case presentation and review of the literature. Annals of Medicine and Surgery.

[7] Fang LC, Lemos B, Douglas J, Iyer J, Nghiem P (2010). Radiation monotherapy as regional treatment for lymph node-positive Merkel cell carcinoma. Cancer.

[8] Chen KT, Papavasiliou P, Edwards K (2013). A better prognosis for Merkel cell carcinoma of unknown primary origin. Am J Surg.

[9] Veness M, Foote M, Gebski V, Poulsen M (2010). The role of radiotherapy alone in patients with merkel cell carcinoma: Reporting the australian experience of 43 patients. Int J Radiat Oncol Biol Phys.

[10] Foote M, Harvey J, Porceddu S, Dickie G, Hewitt S, Colquist S, Zarate D, Poulsen M (2010). Effect of radiotherapy dose and volume on relapse in Merkel cell cancer of the skin. Int J Radiat Oncol Biol Phys.

